# Prevalence and genotype distribution of genital human papillomavirus infection in female sex workers in the world: a systematic review and meta-analysis

**DOI:** 10.1186/s12889-020-09570-z

**Published:** 2020-09-25

**Authors:** Mohammad Farahmand, Mohsen Moghoofei, Abolfazl Dorost, Saeedeh Abbasi, Seyed Hamidreza Monavari, Seyed Jalal Kiani, Ahmad Tavakoli

**Affiliations:** 1 Department of Virology, School of Public Health, Tehran University of Medical Sciences, Tehran, Iran; 2 Department of Microbiology, Faculty of Medicine, Kermanshah University of Medical Sciences, Kermanshah, Iran; 3 Department of Health Economics and Management, School of Public Health, Tehran University of Medical Sciences, Tehran, Iran; 4 Department of Medical Virology, Faculty of Medicine, Iran University of Medical Sciences, Tehran, Iran; 5 Research Center of Pediatric Infectious Diseases, Institute of Immunology and Infectious Diseases, Iran University of Medical Sciences, Tehran, Iran

**Keywords:** Human papillomavirus, HPV, Female sex workers, Prostitution, Epidemiology, Meta-analysis

## Abstract

**Background:**

Female sex workers (FSWs) are amongst the most susceptible groups to acquire human papillomavirus (HPV) infection and consequently, to develop cervical intraepithelial neoplasia and cervical cancer. This is the first systematic review and meta-analysis to provide estimates of the pooled prevalence of HPV infection and the distribution of HPV types among FSWs across the world.

**Methods:**

Five computerized databases were searched for relevant studies published since the inception date of databases to September 2019. The pooled HPV prevalence was calculated by the random effect model described by DerSimonian-Laird. Subgroup analysis was performed to identify the probable sources of heterogeneity. The meta-analysis was performed using the “Metaprop” function in the R package Meta.

**Results:**

Sixty-two studies involving 21,402 FSWs from 33 countries were included in this meta-analysis, and the pooled HPV prevalence was 42.6% (95% confidence interval (CI): 38.5–46.7%). HPV-16 (10.1, 95% CI: 8.2–12.5%), HPV-52 (7.9, 95% CI: 5.9–10.7%), and HPV-53 (6.0, 95% CI: 4.4–8.1%) were the most common high-risk HPV types identified among FSWs. The pooled estimated prevalence of HPV infection among FSWs before and after 2010 were slightly different, 43.6% (95% CI: 36.1–51.4%) and 41.9% (95% CI: 37.2–46.8%), respectively.

**Conclusion:**

Due to the high prevalence of HPV infection, particularly with high-risk types, FSWs have a great susceptibility to the development of cervical and vaginal cancers. Furthermore, they can transmit their infection to their clients, which may result in a high prevalence of HPV and the incidence of HPV-associated malignancies among the general population.

## Background

Human papillomavirus (HPV) is the most frequently sexually transmitted pathogen in humans. There are more than 200 different HPV genotypes recognized to date which are classified into two major groups, high-risk and low-risk genotypes in terms of their malignancy-causing potential [1, 2]. HPV types 6 and 11 are known to be responsible for 90% of genital warts, and types 16 and 18 together cause up to 70% of invasive cervical cancer worldwide. Along with cervical cancer, HPV types 16 and 18 are responsible for 40–50% of invasive vulvar cancer and 70% of vaginal cancer [3]. HPV-16, 18, 31, 33, 35, 39, 45, 51, 52, 56, 58, 59, 66, 68, 73 and 82 are considered high risk genotypes, whereas low-risk genotypes include HPV-6, 11, 34, 40, 42, 43, 44, 54, 61, 70, 72, 81, and 89 [4]. There are three available HPV vaccines licensed by the U.S. Food and Drug Administration (FDA): quadrivalent HPV vaccine, including HPV types 6, 11, 16, and 18 (Gardasil®, produced by Merck); bivalent HPV vaccine, including HPV types 16 and 18 (Cervarix™, produced by GlaxoSmithKline), and nine-valent vaccine, including HPV types 6, 11, 16, 18, 31, 33, 45, 52 and 58 (Gardasil 9; produced by Merck) [5, 6]. Previous reports suggested that 65–100% of the sexually active population are exposed to HPV during their lifespan [7]. Men who have sex with men (MSM) as a sexually active group have a high prevalence of HPV infection (81 and 47% among HIV-positive and HIV-negative MSM, respectively) [8]. The HPV prevalence among women from the general population seems to be lower so that Sabeena et al. have reported the pooled HPV prevalence of 11% among women attending cervical cancer screening clinics [9].

Cervical cancer is the fourth most frequent type of gynecological cancer worldwide, with a high mortality rate. More than 270,000 women annually die from cervical cancer worldwide, which most of them (~ 85%) are in developing countries [10, 11]. Almost all cases of cervical cancer are caused by persistent HPV infection, which is usually transmitted by sexual intercourse. Accordingly, cervical cancer is more common among females with multiple sex partners [12]. In a study conducted by Liu et al., the association between the number of sexual partners and the risk of cervical cancer was assessed through a meta-analysis [13]. Their results suggested that the number of sexual partners was related to the development of cervical cancer.

Female sex workers (FSWs) are a group of females who provide sexual services for economic remuneration [14]. They are a heterogeneous population group who work in varied work environments and community organizations. Street-based sex workers are mainly illegal workers who solicit clients on the street or in public settings such as markets, parks, cinema halls, and service them in alleys, or the clients’ cars. Another group is indoor-based sex workers who are employed to work in brothels, hotels, massage parlors, saunas, and lodges. In some cases, they might solicit clients independently based on both on-street and off-street, by online advertising in newspapers or by phone or text, or might work for a pimp or manager [15–17]. FSWs are at greatly elevated risk of acquiring sexually transmitted infections (STIs), including HPV, and their clients can act as a bridging population toward the general population. The major underlying risk factors for this high-risk group include multiple sex partners [14, 18], unsafe sex behaviors [19, 20], earlier age of sex work debut [21, 22], the years of engaging in sex work [23], and low educational status [24]. It is believed that sexual contact with FSWs contributes to HPV transmission and leads to a high prevalence of cervical cancer in this population [24]. Besides, they elevate the risk of penile cancer in males by the spread of the virus to their male clients [24, 25]. Previous studies have been reported that FSWs have more than two times the probability of having HPV infection compared with women from the general population and have an increased prevalence of abnormal pap smears [26, 27]. It has been reported that FSWs have significantly more cytological abnormalities such as atypical squamous cells of undetermined significance (ASCUS) than women from the normal population. Following abnormal cytology and HPV infection, the occurrence of cervical intraepithelial neoplasia (CIN) 1, CIN 2, or CIN 3 was significantly higher among FSWs compared to the general population [28]. Thus, FSWs are thought to be at elevated risk of cervical dysplasia development due to the high HPV exposure [24, 28, 29].

There are a few review publications focused on the prevalence of HPV infection in the FSW population worldwide. Soohoo et al. reviewed articles assessing the prevalence of HPV types among FSWs in the world [30]. Based on 35 peer-reviewed publications included in their review, the median overall HPV prevalence was 42.7%, with a range of 2.3 to 100%. The ten most common HPV types were HPV-6 (11.5%), 16 (38.9%), 18 (23.1%), 31 (28.4%), 33 (25.0%), 39 (21.6%), 51 (25.0%), 52 (32.7%), 56 (24.0%) and 58 (26.0%). In another study conducted by Peng et al. in 2010, a meta-analytic approach was used to estimate the prevalence and genotype distribution of cervical HPV infection among FSWs in Asia [31]. They found that crude estimates of the cervical prevalence of HPV among 4198 Asian FSWs ranged from 12.8 to 84.8%. According to their results, HPV prevalence among FSWs was nearly 10 times higher than that of the general population of women.

Regarding the important role of HPV infection in the development of cervical cancer and other associated diseases, numerous studies have investigated the prevalence and the distribution of HPV types within FSWs. However, there is not a systematic review and meta-analysis to estimate the overall pooled prevalence of HPV infection in this high-risk population. To the best of our knowledge, this is the first systematic review and meta-analysis that characterizes the global epidemiology of HPV infection and the distribution of high-risk and low-risk HPV types among FSWs. Our study will provide a comprehensive picture of the health status of women engaged in sex trading in the aspect of HPV infection, as one of the major sexually transmitted diseases. The high prevalence of HPV infection among FSWs will persuade policy-makers to promote strategies such as cervical screening and HPV vaccination to reduce the incidence of cervical cancer in this population.

## Methods

This systematic review and meta-analysis was based on the items outlined in the Preferred Reporting Items for Systematic Reviews and Meta-Analyses (PRISMA) guideline [32].

### Search strategy

We conducted an electronic literature search using Web of Science, Scopus, PubMed, Embase, and Google scholar from database inception to September 2019 to identify eligible publications. The details of the search terms for each database are presented in Additional file 1. Moreover, reference lists of all articles included were scanned by hand to find additional eligible studies. All identified records were imported to EndNote software version X8 (Thomson Reuters, California, USA) for further management.

### Selection criteria

In our study, FSWs are defined as women who offer sexual services in return for money, goods, or other markers of economic remuneration. Studies were considered eligible for inclusion if they reported: (1) original data about the prevalence measure for HPV infection among FSWs published in the English language in peer-reviewed journals; (2) the prevalence of HPV DNA in different genital specimens, including cervical, endocervical, vaginal, and cervico-vaginal samples; (3) Studies detecting HPV DNA and transcripts with polymerase chain reaction (PCR), Hybridization, PCR-Hybridization, and Transcription-mediated amplification (TMA) methods; (4) letters to the editor, short communications, and English abstracts with sufficient data. Studies meeting any of the following criteria were excluded: (1) studies estimating the incidence of HPV infection among FSWs; (2) studies of the incidence and the prevalence of HPV infection among transgenders, male sex workers, gays, lesbians, and clients of FSWs; (3) Serological studies that measured antibodies to HPV using methods such as enzyme-linked immunosorbent assay (ELISA); (4) review articles, case reports, posters, and conference abstracts; (5) articles in languages other than English with non-English abstracts.

### Data extraction and quality assessment

Two investigators independently reviewed the eligible articles and extracted data, including the first author’s last name, publication year, study location, total sample size, type of specimen, diagnostic methods, diagnostic indexes, number of HPV-positive cases, and types of HPV. The extracted data were imported into an Excel spreadsheet (Microsoft Corporation, Redmond, WA, USA), and any discrepancies were resolved by a third investigator. A quality assessment of the retrieved studies was performed according to a modified checklist based on the guidelines of the strengthening the reporting of observational studies in epidemiology (STROBE) [33, 34]. The checklist was comprised of 12 questions covering different methodological perspectives. Studies were deemed eligible for the main meta-analysis if they achieved a validity score of at least 8 out of a maximum of 12.

### Statistical analysis

To measure the pooled prevalence of HPV infection among FSWs, a DerSimonian-Laird random-effects meta-analysis was performed [35]. The method is based on the inverse-variance approach, making an adjustment to the study weights dependent on the amount of variation, or heterogeneity, among the different intervention effects. The results of the random-effects and the fixed-effect methods will be identical when there is no heterogeneity between the studies. Where heterogeneity is present, confidence intervals (CIs) around the random-effects summary estimates are wider than CIs around the fixed-effect summary estimates and corresponding claims of statistical significance will be more conservative [36].

To stabilize the variance and normalize their distribution, the logit transformation was used, and the Clopper-Pearson method was applied to estimate the 95% exact CIs for proportions [37]. To explore the possible sources of heterogeneity, subgroup analyses were performed based on study location, type of specimen, diagnostic method, and diagnostic index. To assess the heterogeneity across the included studies, the I^2^ statistic was employed, in which the result is expressed as a percentage [38]. Jackson’s method was used for the estimation of the confidence interval of tau^2 and tau [39]. The meta-analysis was performed using the “Metaprop” function in the R package “meta” [40] (version 3.5.3 [2019-03-11], R Foundation for Statistical Computing, Vienna, Austria). For all statistical tests, differences with *P* values of < 0.05 were considered statistically significant. Graph of the prevalence and genotype distribution of genital HPV was drawn using GraphPad Prism 7.1 for Windows (GraphPad Software, La Jolla California USA).

## Results

### Literature search

**Fig. 1:**
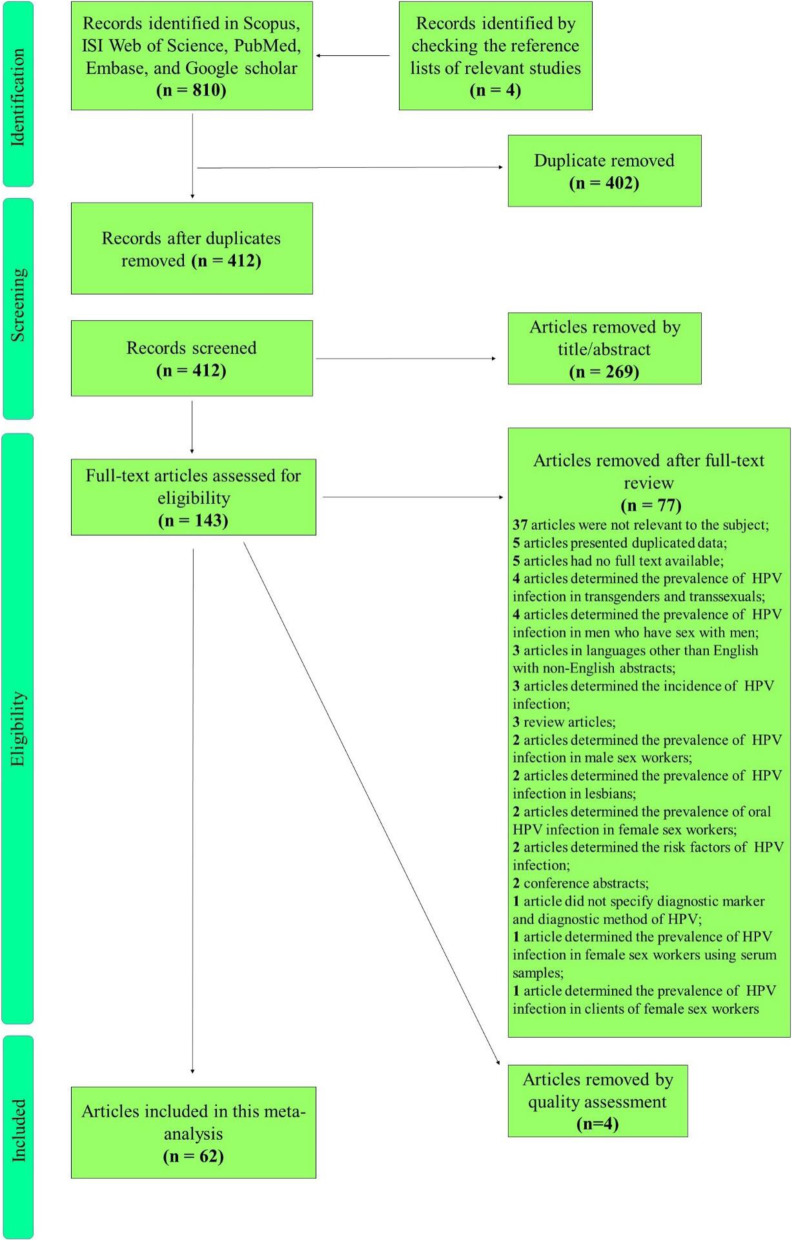
Flowchart presenting the steps of literature search and selection

In the initial literature review, 810 articles were identified through searching the five electronic international databases. Also, 4 relevant articles were found and included by a manual search of the reference lists of the identified articles. A total of 402 duplicates was excluded, and then 412 articles were reviewed by title and abstract, which led to the elimination of 269 articles. The remaining 143 articles were checked for eligibility by the full-text review. After the full-text screening, 77 articles were excluded based on the inclusion/exclusion criteria. Based on the modified STROBE checklist, 62 papers were considered to have good quality (obtained scores of 8 and above), and 4 papers were failed to reach score 8. Overall, 62 articles were included in this systematic review and meta-analysis. Figure 1 shows the process of literature retrieval and screening using a flow diagram.

### Study characteristics

The characteristics of eligible studies in this systematic review and meta-analysis are summarized in Table 1. Sixty-two studies with a total of 21,402 FSWs were included in this meta-analysis. The studies’ publication dates ranged from 1992 to 2019, and they examined the population of 33 countries. The largest study included 2308 and the smallest included 34 FSWs. Most studies investigating the prevalence of HPV infection were from Kenya (*n* = 7) and China (*n* = 4). Out of 62 included studies, 35 assessed the genotype distribution of HPV among FSWs. In one study [57], researchers were investigated the prevalence of only one type of HPV (HPV-16) among FSWs. Therefore, we excluded this study from the main meta-analysis calculating the pooled prevalence to avoid underestimating, and the results of genotype distribution were just included for the analysis. In total, 39.3% of the studies (*n* = 24) were performed before 2010, and 60.7% of the studies (*n* = 37) were performed after 2010.

**Table 1:** The characteristics of all eligible studies in this systematic review and meta-analysis

Author [Ref.]	Publication Year	Study location	Total sample size	No. HPV positive
Velazquez-Hernandez [41]	2019	Mexico	217	12
Diop-Ndiaye [42]	2019	Senegal	436	348
Ferre [43]	2019	Togo	310	140
Lockhart [44]	2019	Kenya	344	97
Adams [14]	2019	Ghana	100	26
Shahesmaeili [45]	2018	Iran	1318	552
Muñoz-Ramírez [46]	2018	Mexico	105	6
Hooi [29]	2018	Curaçao	76	19
Richards [47]	2018	Dominican Republic	143	62
Bui [48]	2018	Cambodia	200	94
Cameron [49]	2018	Kenya	330	97
Nasirian [50]	2017	Iran	99	7
Marra [51]	2017	Netherlands	304	238
Vorsters [52]	2016	Belgium	1334	556
Menon [53]	2016	Kenya	616	357
Singh [54]	2016	India	120	33
Leaungwutiwong [55]	2015	Thailand	100	13
Jia [28]	2015	China	309	191
Gomih-Alakija [56]	2014	Kenya	349	103
Aho [57]	2014	Guinea	223	27
Wang [58]	2013	China	288	192
Patel [59]	2013	Kenya	296	195
Marek [60]	2013	Hungary	34	28
Hoang [61]	2013	Vietnam	281	139
Ersan [24]	2013	Turkey	239	48
Yin [62]	2013	China	802	309
Keten [63]	2013	Turkey	137	53
Li [64]	2012	China	810	315
Ghosh [65]	2012	India	45	35
Couture [66]	2012	Cambodia	220	90
Brown [67]	2012	Peru	199	133
Shikova [68]	2011	Bulgaria	106	46
Matsushita [69]	2011	Japan	196	103
Dal Pogetto [70]	2011	Brazil	102	46
Znazen [71]	2010	Tunisia	188	83
Smith [72]	2010	Madagascar	90	33
Luchters [73]	2010	Kenya	776	429
Rhee [74]	2010	South Korea	2308	939
Valle’s [26]	2009	Guatemala	297	200
Miyashita [75]	2009	Philippines	369	211
del Amo [76]	2009	Spain	549	169
Sultana [77]	2008	Bangladesh	293	222
Sarkar [78]	2008	India	229	58
Hernandez [79]	2008	Vietnam	282	239
Yun [80]	2008	South Korea	188	157
Gazi [81]	2008	Turkey	124	12
Didelot-Rousseau [82]	2006	Burkina Faso	360	238
Chandeying [83]	2006	Thailand	524	120
De Marco [84]	2006	Tunisia	64	28
del Amo [85]	2005	Spain	734	283
Canadas [86]	2004	Spain	187	52
Baay [87]	2004	Belgium	61	19
Mak [88]	2004	Belgium	99	72
Tideman [89]	2003	Australia	288	91
Choi [90]	2003	South Korea	417	194
Juarez-Figueroa [91]	2001	Mexico	495	242
Chan [92]	2001	Singapore	187	27
Kjaer [93]	2000	Denmark	182	59
Ishi [94]	2000	Japan	546	307
Langley [95]	1996	Senegal	681	293
Van Doornum [96]	1993	Netherlands	121	21
Kreiss [97]	1992	Kenya	198	66

*NR* Not reported

### Prevalence of genital HPV infection among FSWs

**Fig. 2:**
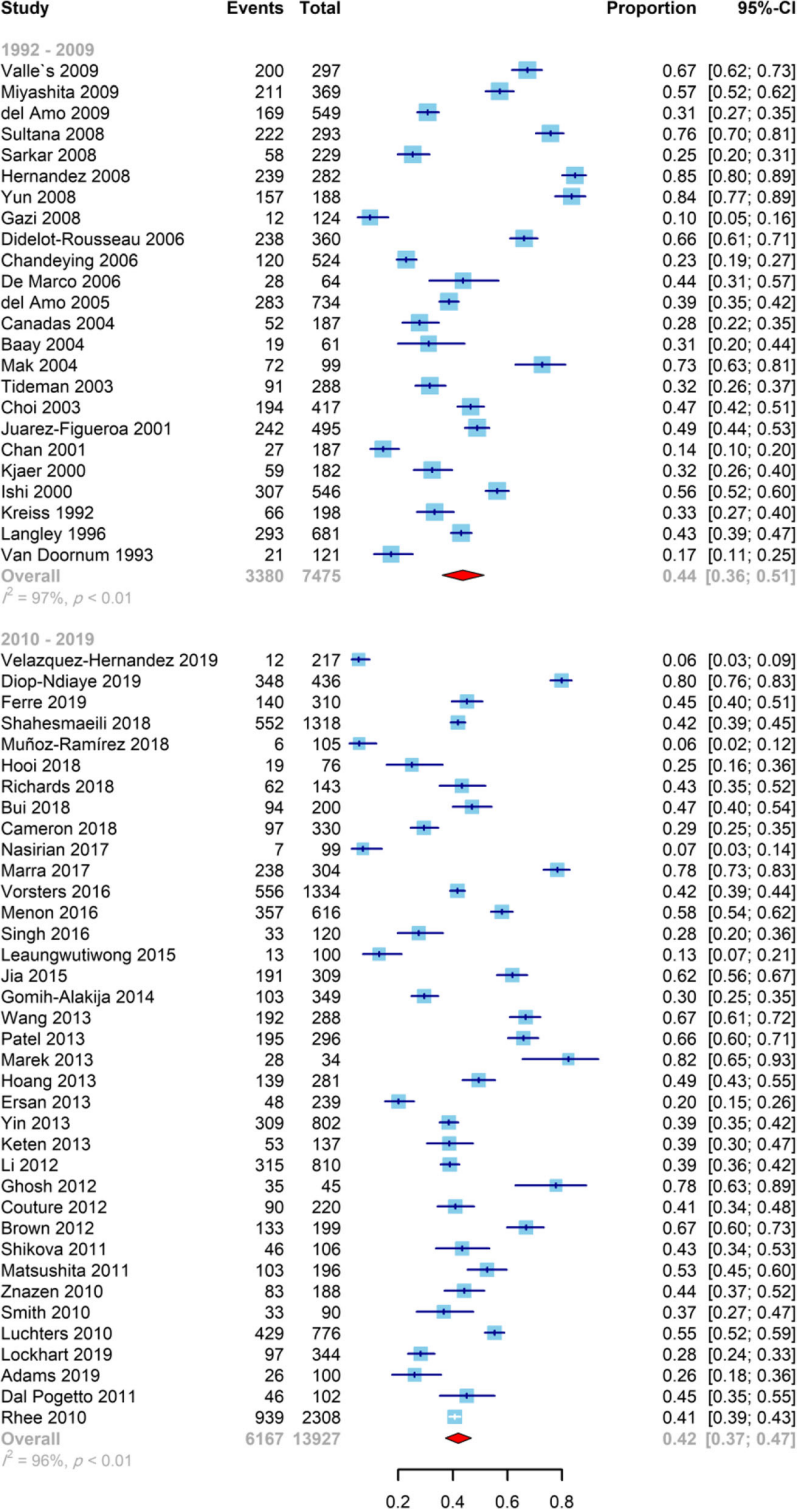
Forest plot of the prevalence of HPV infection in FSWs, stratified by study year (before and after 2010)

Our current study aimed to determine the pooled prevalence of HPV infection in 21,179 FSWs from 32 countries, and the range was from 5.5 to 84.7% of the selected individual studies. Figure 2 shows the prevalence of HPV and 95% CI estimates from individual studies according to the random-effects model. The pooled prevalence of HPV infection among FSWs was 42.6% (95% CI: 38.5–46.7%).

We divided the individual studies into two time periods of publication, before and after 2010. The polled estimated prevalence of HPV infection among FSWs before and after 2010 were slightly different, 43.6% (95% CI: 36.1–51.4%) and 41.9% (95% CI: 37.2–46.8%), respectively (Fig. 2). However, the difference was not statistically significant (*P* = 0.71). Among studies performed after 2010, the maximum and minimum prevalence of HPV infection among FSWs were found in Hungary and Mexico, respectively (82.3, 95%CI: 65.9–91.8% vs 5.5, 95%CI: 3.5–8.7%). The most frequent HPV types detected in Mexico were HPV-73 (5.8, 95%CI: 4.1–8.3%), HPV-39 (5.6, 95%CI: 3.9–8.0%), and HPV-54 (5.2, 95%CI: 3.6–7.6%), while the only one study performed in Hungary [60] did not perform any analysis for HPV typing.

**Fig. 3:**
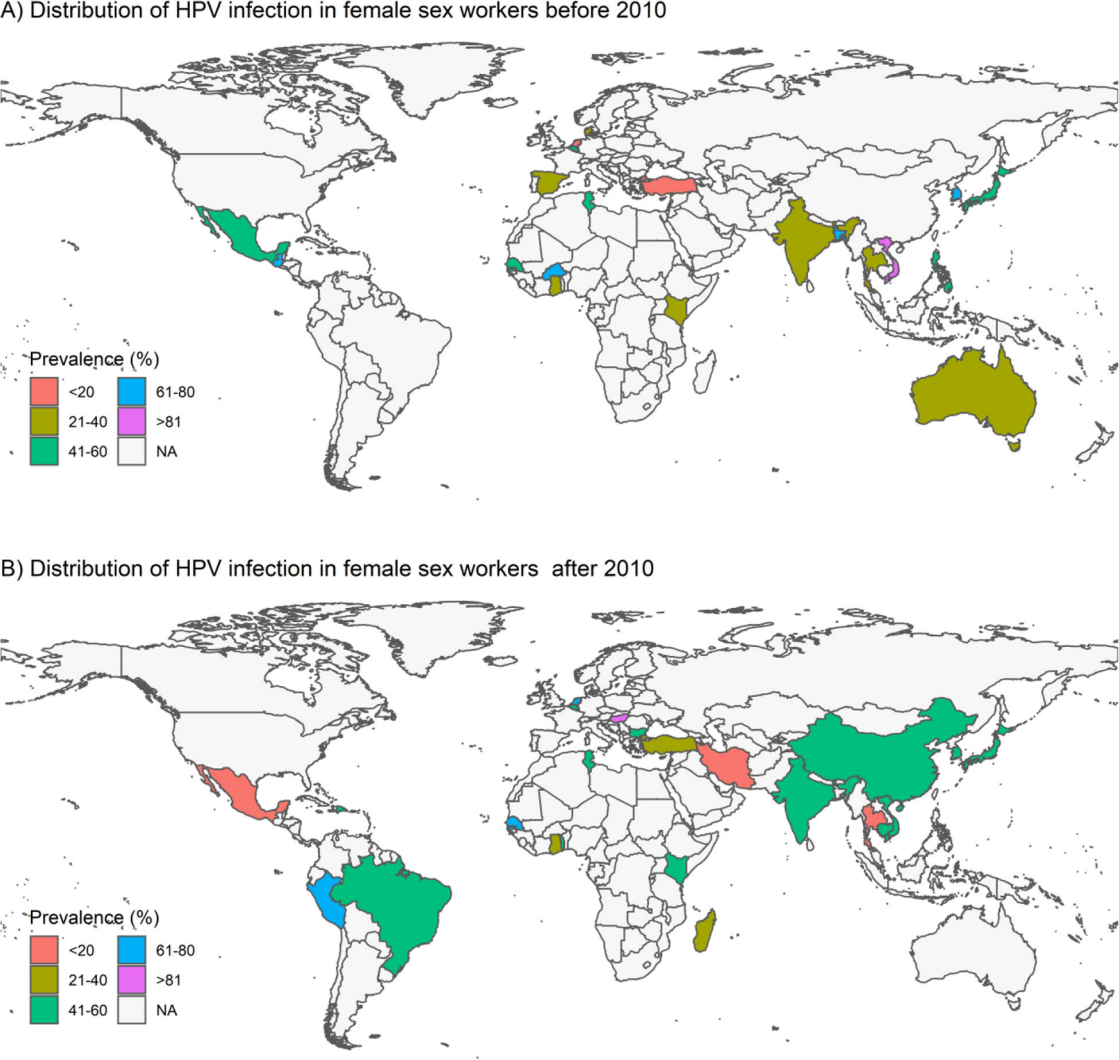
The global maps are presenting the geographical variations in the prevalence of HPV infection in FSWs. Colors indicate the level of prevalence of HPV infection per country

For studies with HPV DNA detection in genital samples of FSWs, there was statistically significant difference between the prevalence of HPV using vaginal (68.4, 95% CI: 37.7–88.5%), cervical (41.9, 95% CI: 37.6–46.4%), cervico-vaginal (46.2, 95% CI: 40.2–52.2%), and endocervical (31.1, 95% CI: 21.1–43.4%) specimens (*P* = 0.04). Concerning HPV detection methods in genital samples of FSWs, PCR, hybridization, PCR-hybridization, and TMA (transcription-mediated amplification) methods were used. The prevalence of HPV was 43.2% (95% CI: 37.8–48.8%), 41.4% (95% CI: 32.4–51.1%), 40.9% (95% CI: 37.7–44.2%), and 31.8% (95% CI: 26.6–37.6%) when PCR-, hybridization-, PCR-hybridization-, and TMA-based methods were used, respectively, and the difference was statistically significant (*P* = 0.02). Table 2 presents more detailed information on the prevalence of HPV infection among FSWs for subgroups. The geographical distribution of HPV infection among FSWs is shown in global maps in Fig. 3.

**Table 2:** Subgroup analysis of the prevalence of HPV infection in female sex workers

Characteristics	Categories	No. of Studies	Pooled prevalence (%) (95% CI)	Heterogeneity test I^**2**^%, ***p***-value	Differences between subgroups; χ^**2**^ test (***p***-value)
**Overall**		61	42.6 (38.5–46.7)	96.9%, *P* < 0.0001	
**Diagnostic method**	PCR	46	43.2 (37.8–48.8)	97.0%, *P* < 0.01	***P*** **= 0.02†**
	Hybridization	7	41.4 (32.4–51.1)	96.2%, *P* < 0.01	
	PCR-Hybridization	3	40.9 (37.7–44.2)	58.4%, *P* = 0.09	
	TMA	4	31.8 (26.6–37.6)	75.0%, *P* < 0.01	
**Sample type**	Cervical	50	41.9 (37.6–46.4)	96.6%, *P* < 0.01	***P*** **= 0.04†**
	Endocervical	4	31.1 (21.1–43.4)	92.1%, *P* < 0.01	
	Vaginal	3	68.4 (37.7–88.5)	99.2%, *P* < 0.01	
	Cervico-vaginal	2	46.2 (40.2–52.2)	0%, *P* = 0.65	
**Diagnostic index**	L1 gene	26	45.1 (38.0–52.3)	96.9%, *P* < 0.01	***P*** **< 0.0001†**
	E6/E7 mRNA transcripts	4	31.8 (26.6–37.6)	75.0%, *P* < 0.01	
	E6 gene	1	75.7 (70.5–80.3)	NA, NA	
	E6/E7 genes	1	26.0 (18.3–35.4)	NA, NA	
**Study year**	1992–2009	24	43.6 (36.1–51.4)	97%, *P* < 0.01	*P* = 0.71
	2010–2019	37	41.9 (37.2–46.8)	97%, *P* < 0.01	

*NA* Not applicable, *PCR* Polymerase chain reaction, *TMA* Transcription-mediated amplification;

† Statistically significant

### Genotype distribution of genital HPV infection in FSWs

**Fig. 4:**
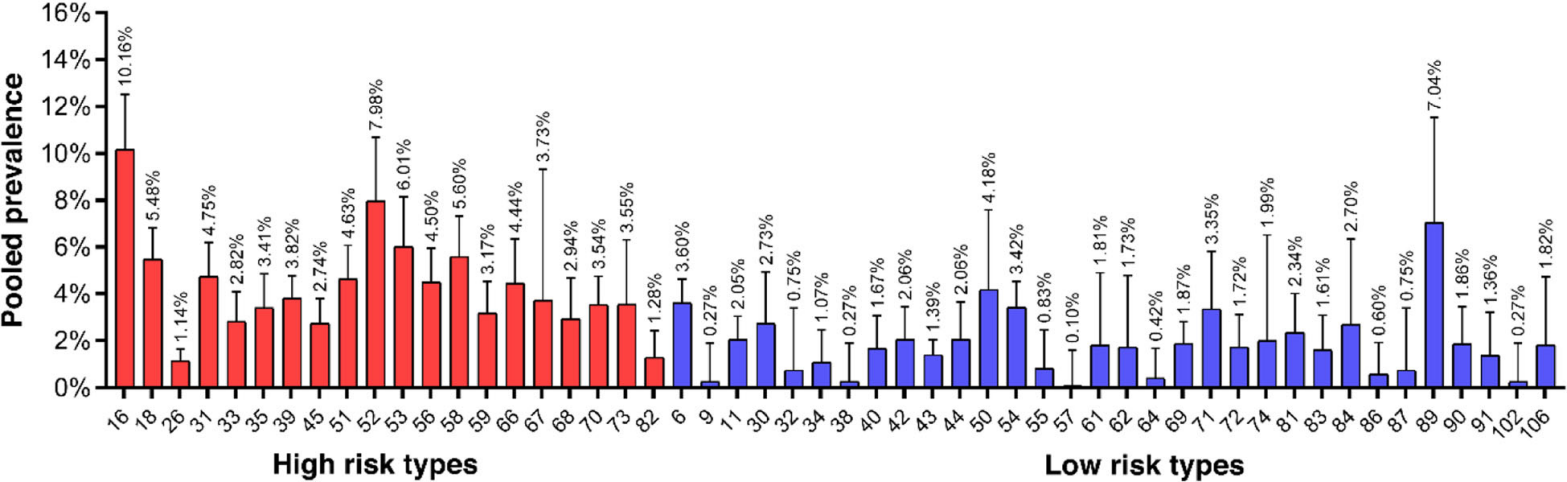
Prevalence and genotype distribution of genital HPV (high-risk and low-risk) among FSWs

Overall, fifty-two HPV types were detected among FSWs across studies. The five most common high-risk HPV types identified were HPV-16 (10.1, 95% CI: 8.2–12.5%), HPV-52 (7.9, 95% CI: 5.9–10.7%), HPV-53 (6.0, 95% CI: 4.4–8.1%), HPV-18 (5.4, 95% CI: 4.4–6.8%) and HPV-58 (5.6, 95% CI: 4.2–7.3%). HPV-89 (7.0, 95% CI: 4.2–11.5%), HPV-50 (4.1, 95% CI: 2.2–7.6%), HPV-6 (3.6, 95% CI: 2.8–4.6%), HPV-54 (3.4, 95% CI: 2.5–4.5%), and HPV-71 (3.3, 95% CI: 1.9–5.8%) were also the most common low-risk HPV types identified among FSWs (Fig. 4).

## Discussion

Sexual intercourse is the main route of transmission of HPV infection, which is known as one of the most common infections around the world. According to this view, FSWs are amongst the most vulnerable group to acquire HPV infection and consequently, to develop precursors of cervical cancer. In part, this arises from the fact that they are constantly being exposed to a large number of risk factors facilitating the spread of sexually transmitted diseases [30]. Previous studies among the general population have reported that the prevalence of HPV ranged from 9 to 13% in the world [98]. As expected, our findings indicated that the number of HPV-positive cases is significantly higher among FSWs compared to the general population, and the prevalence varied from 13 to 82% across the world.

Concerning the overall prevalence and genotype distribution of cervical HPV infection among FSWs, to date, only one meta-analysis has been published by Peng et al. in 2012, which was conducted on 4198 FSWs from nine Asian countries [31]. Their study found a high HPV prevalence in different regions of Asia, so that the overall HPV prevalence in East, South-east, and South Asia were 49.6, 42.9, and 29.3%, respectively. Consistent with this, the results of our meta-analysis also indicated that FSWs in most Asian countries, like Bangladesh, China, Cambodia, India, Japan, Philippines, South Korea, and Vietnam had a prevalence of HPV infection greater than 40%.

The wide range of variations between the different studies can be attributed to differences in the socio-demographic and behavioral characteristics of FSWs. As an example, we found that Mexican FSWs exhibit low levels of HPV infection. It may be related to the implementation of preventive programs such as primary cervical cancer screening, condom promotion, and HPV vaccination, which were effective to reduce the prevalence of HPV infection [41]. The application of vaccines against HPV infection in 11-year-old girls is a part of Mexico’s national immunization program [46]. Previous studies showed that registered FSWs are more likely to engage in screening and prevention programs and more likely to use condoms than clandestine FSWs [99, 100]. Regarding the sex work environment, previous reports indicated that street-based FSWs are at a higher risk for STIs compared to other types of FSWs, mainly due to their social status and engaging in sex work in an extremely unsafe workplace [101, 102]. However, as a limitation, data regarding the sexual behaviors, sex work environments, or type of sex worker did not include in our meta-analysis.

Our results showed that HPV-16 and HPV-52 are the most commonly identified genotypes in FSWs. HPV-16 is considered as the most prominent type involved in the development of cervical cancer and other HPV-associated malignancies. HPV-52 is also an oncogenic HPV type, which is closely related phylogenetically to HPV-16. Previous studies have shown that HPV-52 is the sixth most frequently detected HPV high-risk type in CIN3 and invasive cervical cancer [103].

Similar to Asian countries, the prevalence of HPV positivity was significantly high among European FSWs. Despite high coverage HPV vaccination among females in Europe, our meta-analysis indicated that HPV infection is very common among FSWs in the Netherlands, Belgium, Bulgaria, Denmark, Hungary, and Spain, with a prevalence of HPV infection between 30 and 80%. Our explanation is that vaccination does not protect against HPV types other than 6, 11, 16, and 18. For instance, the majority of FSWs in the Netherlands were infected with types different than what was covered by the current vaccines [51]. To overcome this problem, we recommend the use of a nine-valent vaccine (9vHPV) instead of the bivalent and quadrivalent vaccines. The 9vHPV vaccine contains type 6, 11, 16, 18, 31, 33, 45, 52, and 58 which was approved by the FDA in December 2014, and by the European Medicines Agency (EMA) in June 2015 [104].

Based on the results of the study, we found that the variations in the prevalence of HPV infection in FSWs across the studies could not be explained by the difference in detection methods. This is due to that the detection rates were similar for HPV using PCR, hybridization, and PCR-hybridization, which were applied in 56 (93.3%) studies. However, sample type may be one of the factors leading to differences in prevalence rates. To confirm this finding, our meta-analysis demonstrated that the detection rate of HPV using vaginal samples was significantly higher than cervical, endocervical, and cervico-vaginal samples. We concluded that the vaginal sample is more sensitive for detecting HPV and has a higher level of HPV DNA than the other genital specimens in FSWs. Furthermore, vaginal sampling is a less invasive method and is easily available for all women at the time of a regular HPV test. Owing to the high prevalence of HPV in vaginal samples, vaginal douching with disinfectants after sex with clients seemed to be an effective practice in the reduction of HPV transmission.

In some countries, such as Thailand, Singapore, and Iran, the HPV prevalence is unexpectedly low, and we believe that this is due to several reasons, like limited HPV screening practices, low socioeconomic status, the illegality of sex work, severely limited support systems, unsafe workplaces, fear of stigmatization, and lack of education or skills. Thus, it is so likely that the obtained results in our meta-analysis may not be a precise estimate of the HPV prevalence in these regions.

The present study has some limitations that need to be considered during the interpretation of our results. First, a significant part of the studies investigating the HPV prevalence among FSWs did not perform analysis of HPV genotype distribution, and thus we could not include their results in our meta-analysis of the genotype distribution of HPV. Second, despite the subgroup analyses, significant heterogeneity still existed, suggesting that it arises from other sources that we could not characterize. Finally, there were no published data on the prevalence of HPV infection among FSWs in so many countries such as the United States, Canada, Russia, France, Germany, Italy, the United Kingdom, Nigeria, South Africa, Cameroon, and the Arabian Peninsula.

The HPV vaccination is of public health importance, and since 2009, the World Health Organization (WHO) has recommended the HPV vaccination as a preventive measure against cervical cancer with a primary target population of girls aged 9 to 14 years. As of mid-2019, however, HPV vaccines have been introduced only in 93 countries [105], and many countries still have not implemented national HPV vaccination programs. Governments should consider that HPV vaccination is a cost-effective intervention for the prevention of cervical cancer. Low socioeconomic conditions such as poverty, poor income, and low education levels are often regarded as the main reasons for the involvement of women in sex work. FSWs are a marginalized and stigmatized population across the world and are highly vulnerable to various forms of violence. Previous studies have indicated that the risk of acquiring STIs among FSWs who have experienced violence is nearly three times greater compared to FSWs who have not experienced violence [106]. Criminalization of sex work is associated with more social stigma and increased vulnerability of FSWs to violence [107]. Comprehensive efforts should be directed and prioritized toward reducing violence against this vulnerable population as the vital preventive measures of STIs such as HPV infection.

## Conclusions

In summary, FSWs are a neglected population around the world with a high prevalence of HPV infection, deserving greater attention. Our findings showed that high-risk HPV types are common among FSWs. Persistent infection with high-risk HPV types is the strongest risk factor for the development of cervical intraepithelial neoplasia and cervical or vaginal cancers. Besides, they can transmit their infection to their male clients, which leads to a high HPV prevalence and incidence of HPV-associated malignancies among the general population. Therefore, public health interventions, such as the implementation of national HPV vaccination strategies (particularly by 9vHPV vaccine), regular screening of FSWs for HPV, and encouraging safer-sex strategies like condom use are critical.

## Supplementary information

**Additional file 1.**

## Data Availability

All data generated or analyzed during this study are included in this article.

## Abbreviations

FSWs: Female sex workers
HPV: Human papillomavirus
CI: Confidence interval
STIs: Sexually transmitted Infections
TMA: Transcription-mediated amplification
CIN: Cervical intraepithelial neoplasia
EMA: European Medicines Agency
9vHPV: Nine-valent HPV vaccine
MSM: Men who have sex with men
ASCUS: Atypical squamous cells of undetermined significance
