# Supplementary material for: Prevalence and genotype distribution of genital human papillomavirus infection in female sex workers in the world: a systematic review and meta-analysis
Source: BMC Public Health. 2020 Sep 25;20:1455. doi: 10.1186/s12889-020-09570-z (PMC7519561; doi:10.1186/s12889-020-09570-z)
Supplement: Supplementary file 1 — Additional file 1. [file 12889_2020_9570_MOESM1_ESM.docx]

**Additional file 1**

**PubMed**

("Sex Workers"[Mesh] OR Sex Workers OR Prostitutes OR Prostitute OR Sex Worker OR Sex Worker Clients OR Client, Sex Worker OR Clients, Sex Worker OR Sex Worker Client OR Worker Client, Sex OR Worker Clients, Sex) AND (Human Papilloma Virus OR Human Papilloma Viruses OR Papilloma Virus, Human OR Papilloma Viruses, Human OR Virus, Human Papilloma OR Viruses, Human Papilloma OR HPV, Human Papillomavirus Viruses OR Human Papillomavirus Viruses OR Human Papillomavirus Virus OR Papillomavirus Virus, Human OR Papillomavirus Viruses, Human OR Virus, Human Papillomavirus OR Viruses, Human Papillomavirus OR HPV)

**Embase**

('prostitution'/exp OR 'male prostitution' OR 'prostitution' OR 'sex work') AND ('human papilloma virus' OR 'wart virus' OR 'common wart virus' OR 'condyloma virus' OR 'hpv' OR 'human papillomavirus' OR 'human wart virus' OR 'infectious wart virus' OR 'papilloma virus, human' OR 'papillomavirus, human' OR 'verruca virus' OR 'verruca vulgaris virus' OR 'verruca, viral' OR 'viral verruca' OR 'virus verruca' OR 'virus wart')

**Scopus**

TITLE-ABS-KEY ( ( "Sex Workers" OR prostitutes OR prostitute OR "Sex Worker" OR "Sex Worker Clients" OR "Client, Sex Worker" OR "Clients, Sex Worker" OR "Sex Worker Client" OR "Worker Client, Sex" OR "Worker Clients, Sex" ) AND ("Human Papilloma Virus" OR "Human Papilloma Viruses" OR "Papilloma Virus, Human" OR "Papilloma Viruses, Human" OR "Virus, Human Papilloma" OR "Viruses, Human Papilloma" OR "HPV, Human Papillomavirus Viruses" OR "Human Papillomavirus Viruses" OR "Human Papillomavirus Virus" OR "Papillomavirus Virus, Human" OR "Papillomavirus Viruses, Human" OR "Virus, Human Papillomavirus" OR "Viruses, Human Papillomavirus" ) )

**Web of science**

TS= ( ( "Sex Workers" OR prostitutes OR prostitute OR "Sex Worker" OR "Sex Worker Clients" OR "Client, Sex Worker" OR "Clients, Sex Worker" OR "Sex Worker Client" OR "Worker Client, Sex" OR "Worker Clients, Sex" ) AND ("Human Papilloma Virus" OR "Human Papilloma Viruses" OR "Papilloma Virus, Human" OR "Papilloma Viruses, Human" OR "Virus, Human Papilloma" OR "Viruses, Human Papilloma" OR "HPV, Human Papillomavirus Viruses" OR "Human Papillomavirus Viruses" OR "Human Papillomavirus Virus" OR "Papillomavirus Virus, Human" OR "Papillomavirus Viruses, Human" OR "Virus, Human Papillomavirus" OR "Viruses, Human Papillomavirus" ) )
